# Predicting CAR-T outcomes in R/R DLBCL: a multicenter real-world study of a 5-index model

**DOI:** 10.3389/fimmu.2026.1863194

**Published:** 2026-06-16

**Authors:** Bin Xue, Huina Lu, Yifan Liu, Ying Lu, Wenjun Zhang, Bing Xiu, Xiu Luo, Li Wang, Wenbin Qian, Aibin Liang, Ping Li

**Affiliations:** 1 Department of Hematology, Shanghai Tongji Hospital, Tongji University School of Medicine, Shanghai, China; 2 Clinical Research Ward of Cancer Center, Shanghai Tongji Hospital, Tongji University School of Medicine, Shanghai, China; 3 Department of Hematology, The Affiliated People’s Hospital of Ningbo University, Ningbo, Zhejiang, China; 4 Department of Hematology, Ruijin Hospital Affiliated to Shanghai Jiao Tong University School of Medicine, Shanghai, China; 5 Department of Hematology, The Second Affiliated Hospital, College of Medicine, Zhejiang University, Hangzhou, Zhejiang, China

**Keywords:** CAR-T, DLBCL, multicenter, prediction model, real-world

## Abstract

**Introduction:**

The clinical management of relapsed or refractory diffuse large B‑cell lymphoma (R/R DLBCL) has been transformed by chimeric antigen receptor T‑cell therapy (CAR‑T), yet a significant challenge remains in predicting which patients will derive long‑term benefit.

**Methods:**

This multicenter retrospective real‑world study aimed to validate a previously developed efficacy prediction model for CD19 CAR‑T in Chinese patients with R/R DLBCL. A total of 92 patients with DLBCL who received CD19 CAR‑T across four Chinese centers from August 1, 2021, to November 30, 2024 were included. The 5‑index prediction model (incorporating double‑expressor lymphoma status, TP53 alterations, ECOG performance status ≥2, bulky disease ≥5 cm, and prior therapy lines ≥4) was applied to predict treatment outcomes. The primary endpoints were overall response rate (ORR), complete response (CR) rate, progression‑free survival (PFS), and overall survival (OS).

**Results:**

The median follow‑up was 14.6 months. The C‑index for the 5‑index model was 0.767, indicating good predictive performance. The model effectively stratified patients into different risk groups, with significant differences observed in PFS (P < 0.0001) and OS (P = 0.0007) across groups. The model outperformed traditional prognostic indices such as IPI and R‑IPI.

**Discussion:**

The 5‑index risk model demonstrated robust predictive ability in a real‑world setting, providing a reliable basis for personalized treatment decisions in Chinese DLBCL patients undergoing CAR‑T. Future work will focus on further optimizing the model and conducting multi‑regional validation.

## Introduction

Diffuse large B-cell lymphoma (DLBCL) represents the most common and clinically aggressive form of non-Hodgkin lymphoma, exhibiting significant biological and clinical heterogeneity that complicates therapeutic management (1–3). Although frontline chemoimmunotherapy frequently achieves initial responses, relapsed or refractory (R/R) DLBCL continues to pose substantial treatment challenges. The advent of chimeric antigen receptor T-cell therapy (CAR-T) has transformed the treatment landscape for R/R DLBCL, demonstrating remarkable efficacy in pivotal clinical trials (4). Multicenter real-world studies corroborate these findings, with recent data indicating 12-month overall survival rates of 81.6% and objective response rates reaching 70% among recipients of commercial CD19 CAR-T products (5). However, persistent interpatient variability remains problematic, as 40-60% of patients fail to attain durable remission, highlighting the urgent need for predictive biomarkers to guide patient selection (6, 7).

Current prognostic systems such as the International Prognostic Index (IPI) and revised IPI (R-IPI), developed before the CAR-T era, demonstrate limited utility in predicting outcomes following cellular immunotherapy (8). Novel composite models now incorporate tumor molecular features, particularly TP53 alterations and double-expressor lymphoma (DEL) status alongside clinical variables including tumor bulk and prior treatment lines (9–14). Nevertheless, most models derive from single-center studies or highly selected trial populations, reducing their applicability to broader clinical practice. This constraint is especially evident in Asian populations, where genetic profiles and treatment accessibility often diverge from those in Western cohorts (15, 16). Emerging multi-institutional data demonstrate that the combined presence of DEL and TP53 alterations defines an ultra-high-risk subgroup with dismal CAR-T outcomes, exhibiting 1-year progression-free and overall survival rates approaching 0% despite intensive salvage therapies (17). Conventional prognostic tools fail to account for these molecularly driven resistance mechanisms.

In China, the treatment cost of commercial CAR-T is usually around 150,000 US dollars, and it is not included in the reimbursable drugs of China’s public health care system. The failure of CAR-T without proper evaluation will lead to the loss of both human lives and money. Our previous research sought to address this gap by developing a CD19 CAR-T response prediction model tailored to DLBCL patients (18). The model integrates five clinically relevant factors and assigns one index to each factor: DEL status (MYC/BCL2 co-expression), TP53 alterations, ECOG performance status (≥2), bulky disease (≥5 cm), and prior therapy lines (≥4). The 5-index model based on risk factors can predict DLBCL patients’ responses to CD19 CAR-T: good or poor (0 index: low risk; 1 index: medium risk; 2 index: high risk; 3–5 index: very high risk). While derived from rigorously controlled trial data to reduce variability, external validation in real-world settings remained limited by small cohort sizes. To resolve this constraint, we conducted a large-scale, multicenter validation study using the original framework, enrolling an expanded DLBCL cohort across diverse clinical centers to systematically evaluate the model’s predictive performance and risk stratification in routine practice.

## Methods

This retrospective real-world study of 92 patients with DLBCL who first received CD19 CAR-T was conducted from 4 Chinese centers between August 1, 2021, and November 30, 2024. 37 cases are from Shanghai Tongji Hospital, Tongji University School of Medicine, 22 cases from The Second Affiliated Hospital, College of Medicine, Zhejiang University, 27 cases from Ruijin Hospital Affiliated to Shanghai Jiao Tong University School of Medicine, and 6 cases from The Affiliated People’s Hospital of Ningbo University. The study protocols were approved by the Ethics Committee of Shanghai Tongji Hospital, Tongji University School of Medicine (No. 2021-013-SK-XZ-210410), and adhered to the Declaration of Helsinki. Axi-cel as a commercial CAR-T products was used in 54 cases, Relma-cel was used for another 25 cases, and 13 patients received compassionate use of clinical trial CAR-T product (NCT02537977). Patients who received CAR-T in combination with ASCT as part of the same therapeutic strategy were not included in this study. This exclusion was applied because ASCT involves high-dose chemotherapy and stem-cell rescue and may independently influence treatment response.

The diagnosis of DLBCL based on pathological evaluation according to the 2016 World Health Organization (WHO) classification for tumors of hematopoietic and lymphoid tissues (1). The classification of double-expressor lymphoma (characterized by overexpression of MYC and BCL-2 proteins) and double/triple-hit lymphoma (involving MYC and BCL2 and/or BCL6 rearrangements) followed standard diagnostic criteria (1). The cell of origin (COO) classification, distinguishing between germinal center B-cell (GCB) and non-GCB subtypes, was determined using the Hans algorithm (19). TP53 alterations were identified through mutations detected by next-generation sequencing (NGS) (20) or deletions observed via fluorescence *in situ* hybridization (FISH) analysis, based on the most recent pathological test conducted prior to CAR-T therapy (21).

All CAR T-cell infusions were done during hospitalization. Prior to the infusion of CAR-T therapy (Day 0), FC (Fludarabine 25–30 mg/m²/day + Cyclophosphamide 250–500 mg/m²/day, Day -5 to -3) chemotherapy regimen was administered as preconditioning therapy (22, 23). And FC chemotherapy was given after the drug washout period of bridging therapy (if had). The drug-eluting period between bridging therapy and initiating lymphodepletion for CAR-T therapy strictly adhered to the practice recommendations jointly issued by the European Society for Blood and Marrow Transplantation (EBMT) and the Joint Accreditation Committee of ISCT and EBMT (JACIE) and the European Haematology Association (EHA) (24). CRS and ICANS were assessed and graded according to the consensus guidelines established by the American Society of Transplantation and Cellular Therapy (ASTCT) (25). 78 patients (84.8%) experienced CRS events, 17 patients (18.5%) had sCRS (grade 3-4), and 27 patients (29.3%) developed ICANS. All cases of CRS and ICANS were managed according to the ASTCT and 2022 Chinese consensus guidelines (26) using nonsteroidal anti-inflammatory drugs (NSAIDs), Corticosteroids, and Tocilizumab.

The Lugano classification of 2014 evaluated responses to CD19 CAR T-cell therapy using CT and PET-CT scans (27), categorizing them as complete response (CR), partial response (PR), stable disease (SD), and progressive disease (PD). The overall response rate (ORR) was determined based on the best response (CR or PR) achieved within 3 months after CAR-T cell infusion. To characterize the temporal pattern of treatment failure, response patterns were classified as primary resistance, early relapse, or late relapse. Primary resistance was defined as lack of effective disease control after CAR-T infusion, including insufficient CAR-T expansion or progression/relapse within 3 months (18). Early relapse was defined as progression or relapse occurring more than 3 months but within 6 months after infusion, whereas late relapse was defined as progression or relapse occurring more than 6 months after infusion.

For validation of the 5-index prediction model, treatment efficacy was further dichotomized into good efficacy and poor efficacy according to the early depth and durability of response. Good efficacy was defined as achieving CR within 3 months after CAR-T infusion and maintaining progression-free survival for at least 6 months after infusion. Poor efficacy was defined as failure to achieve CR within 3 months or progression/relapse within the first 6 months after infusion. Thus, the response-pattern categories describe the timing of treatment failure, whereas the good/poor efficacy classification represents the predefined binary composite endpoint used for model validation.

The statistical analysis in this study was conducted primarily via R software (version 4.3.2, Boston, Massachusetts, USA) (28), SPSS software (version 22.0, Chicago, Illinois, USA), and GraphPad Prism software (version 8.0.1). Continuous variables were presented as mean ± standard deviation or median with interquartile range (IQR) based on their normality, assessed by the Shapiro-Wilk test. Group comparisons were conducted using the Student’s t-test (for two groups) or one-way ANOVA (for more than two groups) for normally distributed data, and the Mann-Whitney U test or Kruskal-Wallis test for non-normally distributed data. Categorical variables were expressed as frequencies and percentages and compared using the Chi-square test or Fisher’s exact test, as appropriate. Progression-free survival (PFS) and overall survival (OS) were estimated using the Kaplan-Meier method and compared with the log-rank test. A two-sided p-value < 0.05 was considered statistically significant.

## Results

Patient characteristics at baseline are summarized in **Table 1**. The study concluded follow-up on May 30, 2025, with a median observation period of 14.6 months (IQR: 10.8-19.9). Patients receiving CAR T-cell therapy had a median age of 58.5 years (range: 29-82), with males comprising 55.4% (n=51) of the cohort. Histopathological analysis revealed 65.2% (n=60) non-GCB subtype cases, while 28.3% (n=26) showed double-expression and 7.6% (n=7) demonstrated double/triple-hit characteristics. Extranodal involvement occurred in 41.3% (n=38), with 82.6% (n=76) presenting at Ann Arbor stage III-IV. Bulky disease (maximum tumor diameter ≥5cm) was observed in 30.4% (n=28), and 40.2% (n=37) had IPI scores of 3-5. Treatment history included ≥4 prior lines in 21.7% (n=20), TP53 alterations in 21.7% (n=20), and prior ASCT in 10.9% (n=10). Pre-infusion disease status comprised PR (10.9%, n=10), SD (17.4%, n=16), and PD (71.7%, n=66). Laboratory parameters showed median values of: hemoglobin (HB) 109.0 g/L (IQR: 97.8-123.3), white blood cell (WBC) 3.1×10^9/L (IQR: 2.0-4.2), platelet (PLT) 123.5×10^9/L (IQR: 81.0-172.0), C-reactive protein (CRP) 4.4 mg/L (IQR: 1.6-11.8), lactate dehydrogenase (LDH) 200.0 U/L (IQR: 165.7-259.7), and ferritin 349.5 ng/mL (IQR: 97.8-677.3).

**Table 1:** Baseline table.

Characteristics	Total patients, n= 92
Gender, n (%)	
Male	51 (55.4)
Female	41 (45.6)
Age at enrollment, n (%)	
<60y	35 (51.1)
≥60y	45 (48.9)
Hans classification, n (%)	
GCB	32 (34.8)
N-GCB	60 (65.2)
Double expression, n (%)	
Yes	26 (28.3)
No	66 (71.7)
Double/triple-hit, n (%)	
Yes	7 (7.6)
No	85 (92.4)
Ann Arbor stage, n (%)	
I-II	16 (17.4)
III-IV	76 (82.6)
ECOG before infusion, n (%)	
0-1	46 (50.0)
≥2	46 (50.0)
IPI score at enrollment, n (%)	
0-2	55 (59.7)
3-5	37 (40.2)
Bulky disease, n (%)	
<5 cm	64 (69.6)
≥5 cm	28 (30.4)
Extra-nodual disease, n (%)	
None	54 (58.7)
≥1 organs	38 (41.3)
Prior lines of therapy, n (%)	
1-3	72 (78.3)
≥4 lines	20 (21.7)
TP53, n (%)	
WT	58 (63.0)
Altered	34 (37.0)
Prior ASCT, n (%)	
Yes	10 (10.9)
No	82 (89.1)
The last treatment response, n (%)	
PR	10 (10.9)
SD	16 (17.4)
PD	66 (71.7)
HB g/L, Median [IQR]	109.0 [97.8, 123.3]
WBC*10^9/L, Median [IQR]	3.1 [2.0, 4.2]
PLT*10^9/L, Median [IQR]	123.5 [81.0, 172.0]
CRP mg/L, Median [IQR]	4.4 [1.6, 11.8]
LDH U/L, Median [IQR]	200.0 [165.70, 259.7]
Ferritin ng/mL, Median [IQR]	349.5 [97.8, 677.3]

**Figure 1:**
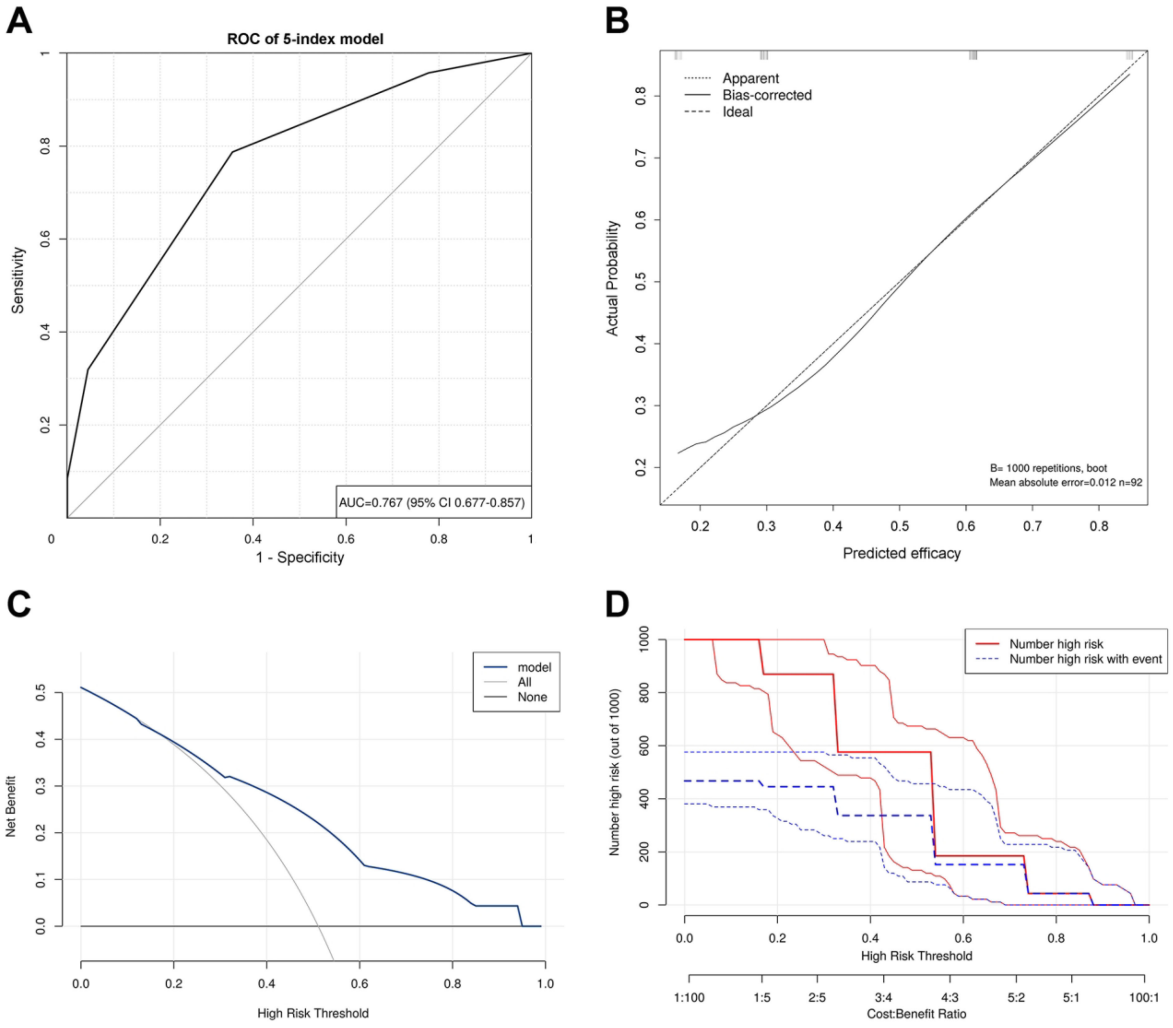
Performance evaluation of the 5-index prediction model. **(A)** Receiver operating characteristic (ROC) curve demonstrating the model’s discriminative ability for predicting CAR-T efficacy (C-index=0.767; 95% CI: 0.677–0.857). **(B)** Calibration curve assessing model fit (nBoots=1000, Hosmer-Lemeshow test: P>0.999; Brier score=0.191). **(C)** Decision curve analysis (DCA) showing clinical utility across risk thresholds (0.2–0.9). **(D)** Clinical impact curve (CIC) validating practical applicability.

The 5-index model made predictions for the entire cohort. With efficacy (good or poor) as the dependent variable, the C-index (AUC) was 0.767 (95% CI: 0.677-0.857) in **Figure 1A**. The Hosmer and Lemeshow goodness of fit test was used to evaluate the fit of the logistic regression model (**Figure 1B**, nBoots=1000), P>0.999. The Brier Score in the calibration curve was 0.191, the Cox-Snell R2 was 0.313 and the Nagelkerke R2 was 0.235. The Decision curve analysis (DCA) revealed that model demonstrated clinical utility across a risk threshold range of 0.2 to 0.9 (**Figure 1C**). The clinical impact curves (CIC) results support that the model is applicable in clinical practice (**Figure 1D**). DCA demonstrated that the model provided a positive net benefit across a wide range of clinically relevant risk thresholds (approximately 20% to 90%), indicating its potential utility for clinical decision-making.

**Figure 2:**
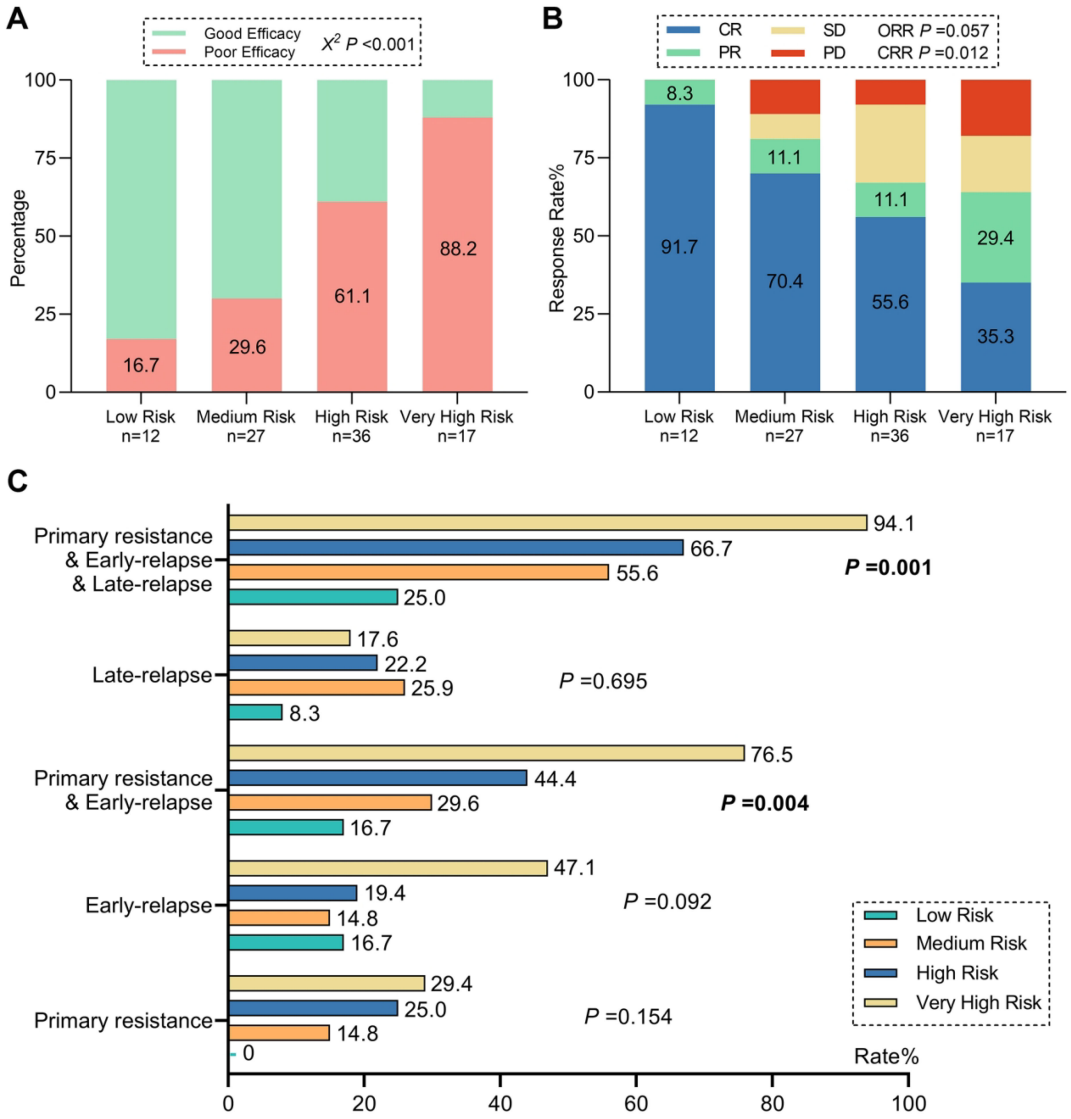
Risk stratification by the 5-index model. **(A)** Significant differences in CAR-T efficacy across risk groups (P<0.001). **(B)** Stratified complete response (CR) rates (CRR, P = 0.012) and objective response rates (ORR, P = 0.057). **(C)** Response patterns: Primary resistance + early relapse (PFS <6 months, P = 0.004) and overall disease recurrence (P = 0.001) showed significant risk stratification. No differences in isolated primary resistance (P = 0.154), early relapse (P = 0.092), or late relapse (P = 0.695).

Over the entire follow-up period, disease relapse occurred in 58 (63.0%) patients and death occurred in 26 (28.3%) patients. The 5-index-based risk model stratified the patients, and the difference in the efficacy of CAR-T was statistically significant (P<0.001, **Figure 2A**). The risk model was also well stratified for the response to CAR-T (**Figure 2B**): the stratification of CR rates showed statistically significant differences (CRR, P = 0.012), and there was a trend in the difference of OR rates (ORR, P = 0.057). In the observation of response patterns, it was found that there were significant statistical differences in risk stratification in primary resistance plus early-relapse (PFS <6 months, P = 0.004), as well as overall disease recurrence (P = 0.001). There was no significant difference in separate grouping of primary resistance (P = 0.154), early-relapse (P = 0.092), and late-relapse (P = 0.695) (**Figure 2C**).

**Figure 3:**
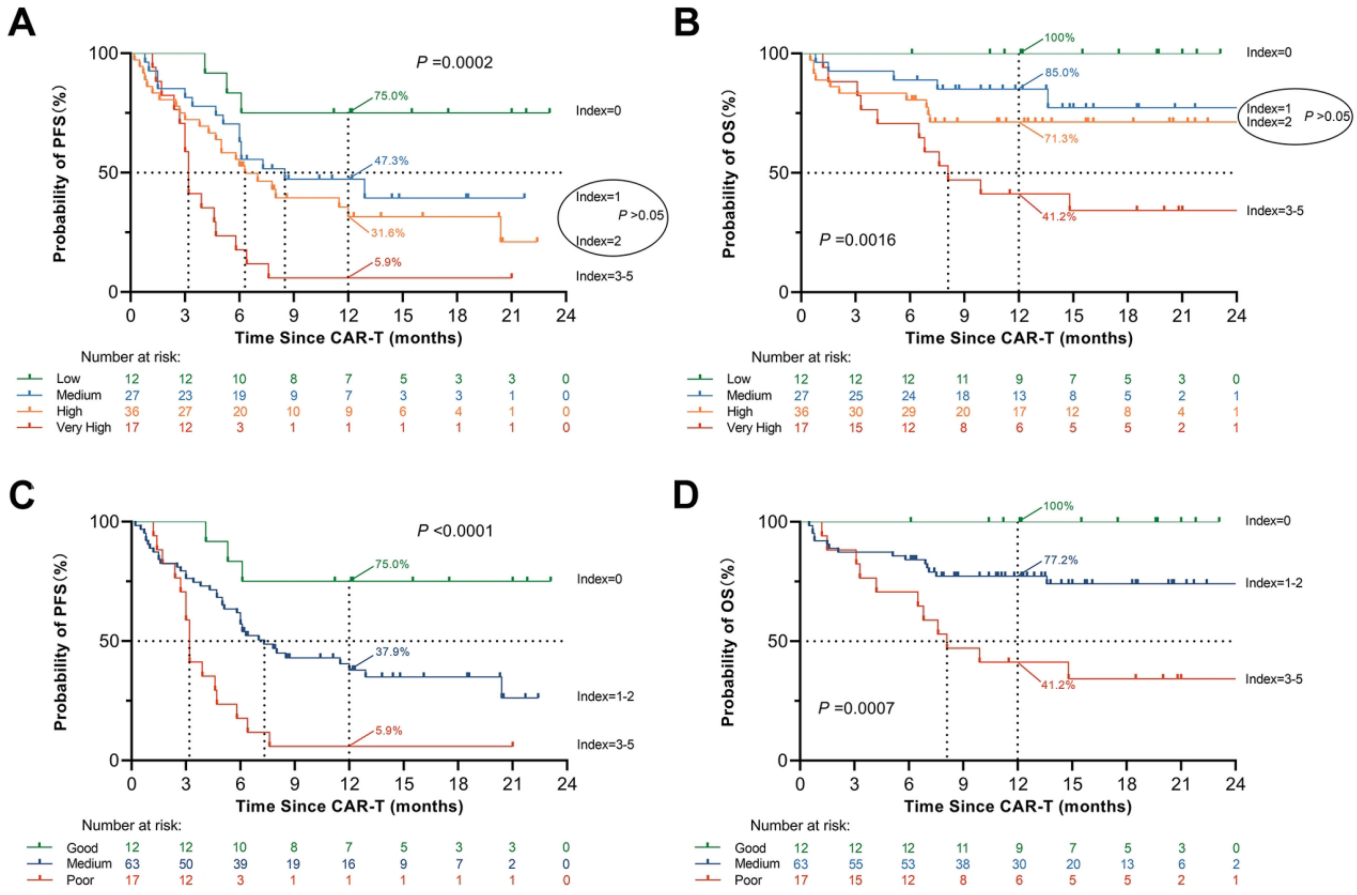
Survival analysis by revised risk groups. **(A)** Progression-free survival (PFS) for initial risk groups (P = 0.0016). **(B)** Overall survival (OS) for initial risk groups (P = 0.0002). **(C)** Revised PFS stratification: Good risk (index=0): 12-month PFS 75.0%; Medium risk (index=1–2): 12-month PFS 37.9% (median PFS 7.3 months); Poor risk (index=3–5): 12-month PFS 5.9% (P<0.0001). **(D)** Revised OS stratification: Good risk: 12-month OS 100%; Medium risk: 12-month OS 77.2%; Poor risk: 12-month OS 41.2% (P = 0.0007).

The degree of dispersion of PFS and OS was verified using the risk model. Significant differences were observed in the K-M curves for PFS (P = 0.0016, **Figure 3A**) and OS (P = 0.0002, **Figure 3B**) between the two groups. However, we observed that the differences in PFS and OS between the medium risk group (index=1) and the high risk group (index=2) were not significant. So we evaluated the risk model revised according to the degree of prognostic dispersion and grouped index=1 and index=2 into the same group (medium). The median PFS of this group of patients was 7.3 months (95% CI, 6.0 to NR), and the median OS was not achieved. The good(index=0), medium(index=1-2), and poor groups(index=3-5) of revised risk model, whose PFS reached 12 months were 75.0%,37.9% and 5.9% (**Figure 3C**). The proportions of achieving a 12-month OS were 100%,77.2%, and 41.2%, respectively (**Figure 3D**).

**Table 2** shows the baseline among the three independent prognostic groups. In addition to the baseline factors in the 5-index model, the statistically different factors include gender, pre-infusion HB and PLT. Further univariate logistic regression analysis was conducted on gender, pre-infusion HB and PLT as independent variables, and the dependent variable was efficacy of CAR-T (**Supplementary Table 1**). After taking the five factors (double expression, TP53, ECOG, bulky disease, prior lines of therapy) as adjustment variables, gender, pre-infusion HB and PLT had no significant correlation with the efficacy.

**Table 2:** Adjusted baseline analyses.

Characteristics	R-low risk, n=12	R-medium risk, n=63	R-high risk, n=17	P
Gender, n (%)				0.025
Male	11 (91.7)	32 (50.8)	8 (47.1)	
Female	1 (8.3)	31 (49.2)	9 (52.9)	
Age at enrollment, n (%)				0.505
<60y	8 (66.7)	31 (49.2)	8 (47.1)	
≥60y	4 (33.3)	32 (50.8)	9 (52.9)	
Hans classification, n (%)				0.798
GCB	5 (41.7)	22 (34.9)	5 (29.4)	
N-GCB	7 (58.3)	41 (65.1)	12 (70.6)	
Double expression, n (%)				<0.001
Yes	0 (0)	12 (19.0)	14 (82.4)	
No	12 (100)	51 (81.0)	3 (17.6)	
Double/triple-hit, n (%)				0.484
Yes	1 (8.3)	6 (9.5)	0 (0)	
No	11 (91.7)	57 (90.5)	17 (100)	
Ann Arbor stage, n (%)				0.229
I-II	0 (0)	13 (20.6)	3 (17.6)	
III-IV	12 (100)	50 (79.4)	14 (82.4)	
ECOG before infusion, n (%)				<0.001
0-1	12 (100)	31 (49.2)	3 (17.6)	
≥2	0 (0)	32 (50.8)	14 (82.4)	
IPI score at enrollment, n (%)				0.122
0-2	6 (50.0)	42 (66.7)	7 (41.2)	
3-5	6 (50.0)	21 (33.3)	10 (58.8)	
Bulky disease, n (%)				0.002
<5 cm	12 (100)	45 (71.4)	7 (41.2)	
≥5 cm	0 (0)	18 (28.6)	10 (58.8)	
Extra-nodual disease, n (%)				0.123
None	4 (33.3)	38 (60.3)	12 (70.6)	
≥1 organs	8 (66.7)	25 (39.7)	5 (29.4)	
Prior lines of therapy, n (%)				0.007
1-3	12 (100)	51 (81.0)	9 (52.9)	
≥4 lines	0 (0)	12 (19.0)	8 (47.1)	
TP53, n (%)				0.005
WT	12 (100)	38 (60.3)	8 (47.1)	
Altered	0 (0)	25 (39.7)	9 (52.9)	
Prior ASCT, n (%)				>0.999
Yes	11 (91.7)	56 (88.9)	15 (88.2)	
No	1 (8.3)	7 (11.1)	2 (11.8)	
The last treatment response, n (%)				0.508
PR	0 (0)	8 (12.7)	2 (11.7)	
SD	3 (25.0)	12 (19.0)	1 (5.9)	
PD	9 (75.0)	43 (68.3)	14 (82.4)	
HB g/L, Median [IQR]	121.5 [115.3, 127.5]	110.0 [97.0, 124.5]	101.0 [90.0, 107.0]	0.002
WBC*10^9/L, Median [IQR]	2.6 [1.8, 3.5]	3.1 [2.1, 4.2]	3.0 [2.4, 4.4]	0.649
PLT*10^9/L, Median [IQR]	125.5 [71.5, 153.0]	135.0 [89.0, 178.5]	93.0 [59.0, 123.0]	0.047
CRP mg/L, Median [IQR]	4.3 [2.9, 5.2]	4.1 [1.0, 11.7]	10.3 [3.3, 20.9]	0.257
LDH U/L, Median [IQR]	191.7 [176.6, 217.3]	201.0 [156.5, 260.2]	228.0 [175.0, 351.0]	0.343
Ferritin ng/mL, Median [IQR]	459.0 [330.0, 611.7]	267.5 [55.8, 605.4]	458.5 [321.0, 826.0]	0.105

Response rates among the various products of Axi-cel, Relma-cel, and compassionate CAR-T groups were comparable, with no significant intergroup differences observed in overall response rate or complete response rate (ORR P = 0.395, CRR P = 0.661, **Supplementary Table 2**). Similarly, CD19 CAR-T response patterns showed no significant variation (P>0.05).

**Figure 4:**
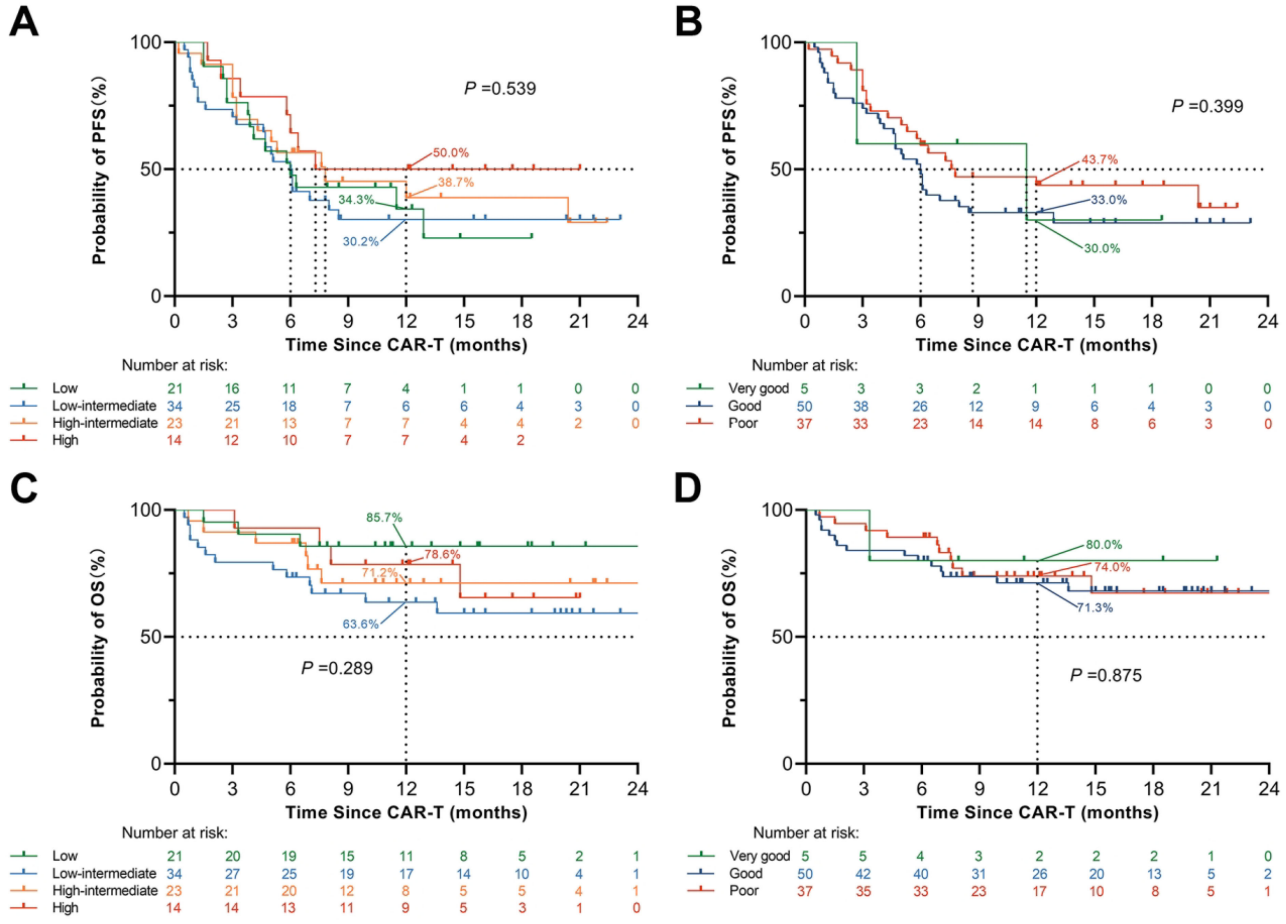
Comparison with conventional prognostic indices. **(A)** PFS by International Prognostic Index (IPI; P = 0.539). **(B)** PFS by revised IPI (R-IPI; P = 0.399). **(C)** OS by IPI (P = 0.289). **(D)** OS by R-IPI (P = 0.875). All IPI/R-IPI risk groups had ≤50% 12-month PFS and >50% 12-month OS, with no significant stratification.

The International Prognostic Index (IPI) and the revised International Prognostic Index (R-IPI) have been used for the prognostic assessment of diffuse large B-cell lymphoma (DLBCL) to present discrete outcome groupations. However, IPI (P = 0.539, **Figure 4A**) and R-IPI (P = 0.399, **Figure 4B**) had no significant meaning in the classification of PFS, and the 12-month PFS of all risk groups did not exceed 50%. Similarly, in the classification of OS, the 12-month OS of all risk groups in IPI (P = 0.289, **Figure 4C**) and R-IPI (P = 0.875, **Figure 4D**) exceeded 50%, with no statistical difference.

## Discussion

With CAR-T as one of the second-line and third-line plans for DLBCL patients in China (and even as a first-line option for high-risk patients), the need for population-specific prediction tools for clinicians. This real-world analysis assessed a five-factor risk model incorporating double expression, TP53 alterations, ECOG ≥2, bulky disease ≥5 cm, and ≥4 prior-line therapies, demonstrating superior prognostic discrimination for CAR-T outcomes compared to conventional IPI/R-IPI indices in stratifying PFS and OS. The model achieved reliable performance (C-index: 0.767; Brier score: 0.191) of efficacy prediction, with decision curve analysis confirming clinical utility across a broad risk threshold range (0.2-0.9). Recalibration by merging medium- and high-risk groups further enhanced prognostic precision, revealing marked disparities in 12-month PFS (P<0.0001) and OS (P = 0.0007) between good-, medium- and poor-risk cohorts.

Our findings support recent evidence underscoring IPI/R-IPI limitations in the CAR-T era (29). Neither index significantly stratified PFS or OS in this cohort, with 12-month PFS rates ≤50% across all IPI groups. This discrepancy likely stems from IPI’s reliance on pre-chemotherapy variables irrelevant to CAR-T biology, such as Ann Arbor stage or extranodal involvement. In contrast, the five-factor model prioritizes post-chemotherapy resistance mechanisms (e.g., TP53 mutations, high pretreatment lines) and CAR-T-specific challenges (e.g., tumor bulk as a CRS driver).

Beyond validating the five-factor model, we explored additional prognostic variables, including gender and pre-infusion hemoglobin (HB) and platelet (PLT) levels. Univariate logistic regression revealed no significant association between these factors and CAR-T efficacy after adjusting for the core model (30, 31), which may be due to the limited sample size. These variables may indirectly reflect tumor aggressiveness or bone marrow reserve rather than directly influencing CAR-T expansion or persistence (32, 33). The future to further expand sample size research will focus on the impact of these indicators.

Comparative analyses of commercial CAR-T efficacy in R/R DLBCL remain constrained by the lack of randomized trials. A propensity score-matched analysis of the French DESCAR-T registry (N = 809) reported divergent outcomes: tisa-cel recipients achieved an ORR of 52% (CR: 40%) with median OS of 12 months, while axi-cel showed superior efficacy (ORR: 83%; CR: 58%; median OS: 26 months) (34). But tisa-cel has not entered commercial use in China. In our cohort, response rates across CAR-T products (axi-cel, relma-cel, and compassionate-use constructs) were comparable, with no significant differences in ORR or CRR, suggesting patient-specific factors may outweigh product selection. Further studies are needed to explore potential interactions between CAR-T products and patient characteristics.

Consistent with prior research, TP53 alterations (21.7% of our cohort) drive genomic instability and chemoresistance, potentially synergizing with CAR-T mechanisms. Similarly, double-expressor lymphoma status (28.3%), linked to MYC/BCL-2 dysregulation, may exacerbate tumor proliferation and immune evasion. These biologic factors, combined with clinical markers of aggressive disease (e.g., bulky tumors, high pretreatment burden), define a risk landscape poorly captured by traditional indices. Notably, the model retained predictive power despite heterogeneous bridging therapies and CAR-T products, supporting broad applicability. Our study provides critical insights into the temporal dynamics of CAR-T failure in DLBCL. While current understanding of relapse risk factors remains incomplete, we systematically validated that our model achieves exceptional risk stratification for early relapse (<6 months) (18, 35). The time-dependent patterns suggest distinct biological mechanisms governing early versus late recurrence, with immediate failures reflecting intrinsic tumor resistance and later events relating to CAR-T functional decline (36, 37).

Our study advances CAR-T prognostication by validating the model in real-world cohorts, including compassionate-use recipients, patients with a median age of 58.5 years, and heavily pretreated cases (21.7% with ≥4 prior lines). The proposed risk stratification system balances clinical practicality with accuracy. However, limitations include the retrospective design, potential selection bias, and a median follow-up (14.6 months) insufficient for long-term assessment. While the sample size was adequate for model validation, larger studies are needed to confirm these findings and evaluate generalizability beyond Chinese populations. International collaborations could further refine the model’s applicability.

## Conclusion

In conclusion, our study provides valuable insights into the efficacy prediction of CAR-T therapy for Chinese DLBCL patients. The 5-index risk model has been shown to be a robust tool for stratifying patients into different risk groups and predicting treatment outcomes. By validating this model in a multicenter retrospective real-world study, we have confirmed its prognosis utility and provided a reliable basis for personalized treatment decisions. The model will be further optimized and multi-regional validation will be carried out in the future.

## Data Availability

The raw data supporting the conclusions of this article will be made available by the authors, without undue reservation.
