# Supplementary material for: Predicting CAR-T outcomes in R/R DLBCL: a multicenter real-world study of a 5-index model
Source: Front Immunol. 2026 Jun 16;17:1863194. doi: 10.3389/fimmu.2026.1863194 (PMC13314439; doi:10.3389/fimmu.2026.1863194)
Supplement: Supplementary file 1 [file Table1.docx]

Supplementary Table 1. Univariate analysis of efficacy (adjusted)

| **Characteristics** | **OR** | **95%CI** | ***P*** | **adjusted OR** | **adjusted 95%CI** | **adjusted *P*** |
| --- | --- | --- | --- | --- | --- | --- |
| **Gender (n)** |  |  |  |  |  |  |
| Male (51) vs. Female (41) | 0.40 | (0.17, 0.94) | 0.036 | 0.43 | (0.15, 1.24) | 0.119 |
| **HB g/L, n=92** | 1.00 | (0.99, 1.00) | 0.407 | 1.00 | (0.99, 1.01) | 0.633 |
| **PLT*10^9/L, n=92** | 0.98 | (0.95, 1.00) | 0.042 | 0.99 | (0.97, 1.02) | 0.613 |

Supplementary Table 2. Response among various CART products

| **Characteristics** | **Axi-cel, n=54** | **Relma-cel, n=25** | **Compassionate CART, n=13** | ***P*** |
| --- | --- | --- | --- | --- |
| Response, N (%) |  |  |  |  |
| CR | 36 (66.7) | 13 (52.0) | 7 (53.8) | 0.395 |
| PR | 6 (11.1) | 5 (20.0) | 2 (15.4) |  |
| SD | 8 (14.8) | 5 (20.0) | 1 (7.7) |  |
| PD | 4 (7.4) | 2 (8.0) | 3 (23.1) |  |
| OR (CR+PR) | 42 (77.8) | 18 (72.0) | 9 (69.2) | 0.661 |
| Primary resistance, N (%) |  |  |  | 0.573 |
| Yes | 11 (20.4) | 6 (24.0) | 1 (7.7) |  |
| No | 43 (79.6) | 19 (76.0) | 12 (92.3) |  |
| Early-relapse, N (%) |  |  |  | 0.495 |
| Yes | 10 (18.5) | 7 (28.0) | 4 (30.8) |  |
| No | 44 (81.5) | 18 (72.0) | 9 (69.2) |  |
| Primary resistance+ Early-relapse, N (%) |  |  |  | 0.522 |
| Yes | 21 (38.9) | 13 (52.0) | 5 (38.5) |  |
| No | 33 (61.1) | 12 (48.0) | 8 (61.5) |  |
| Late-relapse, N (%) |  |  |  | 0.709 |
| Yes | 13 (24.1) | 4 (16.0) | 2 (15.4) |  |
| No | 41 (75.9) | 21 (84.0) | 11 (84.6) |  |
| Primary resistance+ Early-relapse+ Late-relapse, N (%) |  |  |  | 0.715 |
| Yes | 34 (63.0) | 17 (68.0) | 7 (53.8) |  |
| No | 20 (37.0) | 8 (32.0) | 6 (46.2) |  |
